# Nutrient niche specificity for glycosaminoglycans is reflected in polysaccharide utilization locus architecture of gut *Bacteroides* species

**DOI:** 10.3389/fmicb.2022.1033355

**Published:** 2022-11-29

**Authors:** Annelieke Overbeeke, Bela Hausmann, Georgi Nikolov, Fatima C. Pereira, Craig W. Herbold, David Berry

**Affiliations:** ^1^Centre for Microbiology and Environmental Systems Science, University of Vienna, Vienna, Austria; ^2^Doctoral School in Microbiology and Environmental Science, University of Vienna, Vienna, Austria; ^3^Joint Microbiome Facility, Medical University of Vienna, Vienna, Austria; ^4^Department of Laboratory Medicine, Medical University of Vienna, Vienna, Austria

**Keywords:** hyaluronan, chondroitin sulfate, glycosaminoglycans, bacteroides, intestine

## Abstract

**Introduction:**

Glycosaminoglycans (GAGs) present in the mucosal layer can be used as nutrients by certain intestinal bacteria, particularly members of the Bacteroides. GAG abundances are altered in some diseases such as inflammatory bowel diseases, which may affect microbial composition and activity, and it is therefore important to understand GAG utilization by members of the gut microbiota.

**Methods:**

We used growth assays, transcriptomics, and comparative genomics to evaluate chondroitin sulfate (CS) and hyaluronan (HA) degradation ability by multiple gut Bacteroides species.

**Results and discussion:**

We found that not all Bacteroides species able to degrade CS could also degrade HA, despite having lyases which act on both compounds. We propose that in the model organism Bacteroides thetaiotaomicron, the lyase BT_3328 in combination with surface binding proteins BT_3329 and BT_3330 and potentially BT_4411 are involved in HA breakdown. Furthermore, degradation of both compounds provides public goods for other Bacteroides, including non-degraders, suggesting that cooperative degradation as well as cross-feeding may be widespread in the mucosal glycan utilization clade.

## Introduction

**Figure 1:**
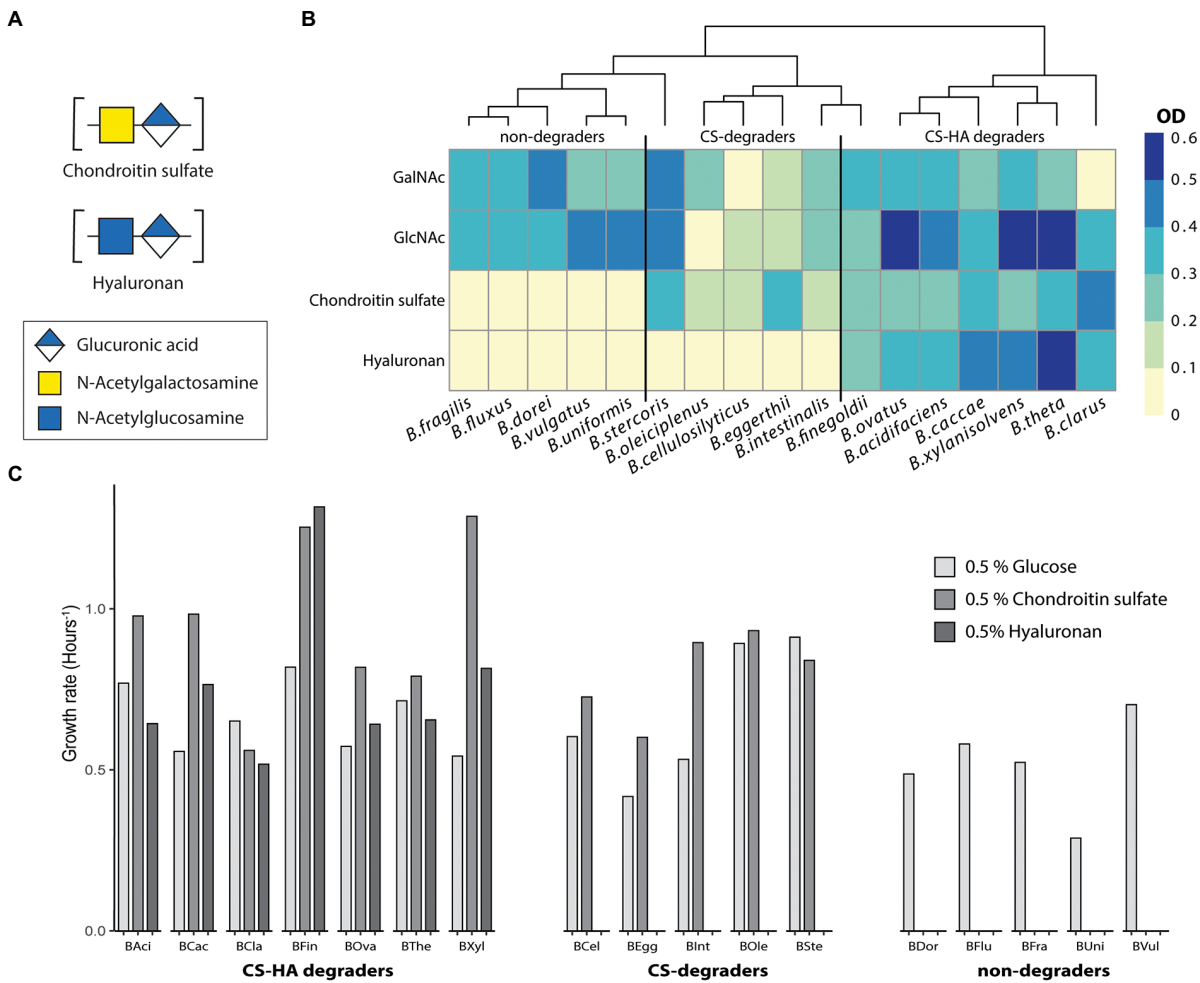
Not all strains capable of degrading CS can also degrade HA. Growth metrics for 17 *Bacteroides* species on 0.5% of glucose, chondroitin sulfate (CS) or hyaluronan (HA). All growth was performed in triplicates with at least two biological replicates. **(A)** Average final optical density (OD_600_) at 19 h for 17 *Bacteroides* strains. Almost all species can degrade GalNAc and GlcNAc whereas there are distinct groups for those which can degrade both chondroitin sulfate (CS) and hyaluronan (HA), CS-only or neither. OD < 0.1 was considered to be no growth. **(B,C)** Growth rate (hours^−1^) measured from the exponential part of the growth curve. BAci, *B*. *acidifaciens*; BCac, *B*. *caccae*; BCel, *B*. *cellulosilyticus*; BCla, *B*. *clarus*; BDor, *B*. *dorei*; BEgg, *B*. *eggerthii*; BFin, *B*. *finegoldii*; BFlux, *B*. *fluxus*; BFra, *B*. *fragilis*; BInt, *B*. *intestinalis*; Bole, *B*. *oleiciplenus*; BOva, *B*. *ovatus*; BSte, *B*. *stercoris*; BThe, *B*. *thetaiotaomicron*; BUni, *B*. *uniformis*; BVul, *B*. *vulgatus*; BXyl, *B*. *xylanisolvens*.

The human gastrointestinal tract is lined with a layer of secreted mucus that varies in thickness and composition (Pelaseyed et al., 2014). Not only does mucus protect epithelial cells from invasion by pathogens, it also harbors commensal and potentially-beneficial bacteria (Pelaseyed et al., 2014; Sato et al., 2020; Yuan et al., 2020; Martin-Gallausiaux et al., 2021). Beneath the secreted mucus layer there is another defense barrier for the epithelial cells, namely the glycocalyx. This is a thin layer directly covering the epithelial cells consisting of glycoproteins and glycolipids (Moran et al., 2011). Proteoglycans are a subclass of glycoproteins that have glycosaminoglycans (GAGs) attached to a protein backbone. Two predominant intestinal GAGs are chondroitin sulfate (CS) and hyaluronan (formerly known as hyaluronic acid, HA). CS is made up of disaccharides containing glucuronic acid (GlcA) and N-acetyl-galactosamine (GalNAc) and, as the name suggests, can be sulfated on multiple positions (Figure 1A). HA consists of repeating monomers of GlcA and N-acetyl-glucosamine (GlcNAc; Bartlett and Park, 2010). Similar to the secreted mucus layer, there is a constant turn-over of the glycocalyx, resulting in breakdown products that are released into the mucus, thus supplying bacteria in the gut lumen with additional potential nutrient sources (Moran et al., 2011). GAGs are not only an important microbial nutrient source in the healthy gut, but also in intestinal inflammatory diseases such as ulcerative colitis, where they have been shown to be enriched in the intestinal mucosa (Belmiro et al., 2005). Bacteria capable of degrading GAGs have been shown to proliferate during colitis development (Lee et al., 2009), and it is thus vital to have a better understanding of GAG metabolism by intestinal bacteria. Additionally, hyaluronan supplements have been intensively studied for symptom release in osteoarthritis, inflammation and necrotizing enterocolitis (Asari et al., 2010; Hewlings et al., 2019; Chaaban et al., 2021).

Bacteroidota are one of the main phyla in the intestinal tract (Eckburg et al., 2005) and are capable of degrading both dietary as well as host-derived glycans (Salyers et al., 1977). Enzymes required for glycan degradation include glycoside hydrolases and polysaccharide lyases, which are organized on the genome into polysaccharide utilization loci (PULs; McKee et al., 2021). *Bacteroides thetaiotaomicron* (*B*. *theta*) is one of the best characterized gut commensal bacteria and is a widely used model organism to study bacterial physiology and microbe-host interactions. In *B*. *theta*, degradation of CS and HA is mediated by PUL 57 (Martens et al., 2008; Raghavan et al., 2014). Within this PUL, three periplasmic lyases have been identified for CS-HA degradation (BT_3324, BT_3350 and BT_4410), with two having a higher affinity for CS (BT_3324 and BT_3350) and the third (BT_4410) having a higher affinity for HA (Raghavan et al., 2014). Additionally, a novel surface lyase with a high affinity for CS (BT_3328) has recently been identified (Ndeh et al., 2018, 2020). Although *B*. *theta* PUL architecture for CS and HA degradation has been well-studied, the ability of other gut *Bacteroides* to use these compounds and the conservation of this PUL among the gut *Bacteroides* remains less well understood.

The concept of “public goods” is an essential aspect of commensal and mutualistic interactions in the gut microbiota. Some bacteria can promote the growth of others by providing them with breakdown products of more complex compounds that they themselves cannot degrade (Rakoff-Nahoum et al., 2014; Banerjee et al., 2018). In marine environments, Bacteroidota have been suggested to be largely selfish utilizers of polysaccharides, meaning that they use cell-surface enzymes for degrading large polysaccharides and directly transport breakdown products into the periplasm (Reintjes et al., 2019). In the gut, it has been shown that some members of the Bacteroidales such as *B*. *theta* and *Bacteroides ovatus* provide public goods during degradation of the dietary polysaccharides levan and inulin to non-degrading bacteria. Additionally, breakdown products for the same polysaccharide can differ depending on the species responsible for degradation (Rakoff-Nahoum et al., 2014). This potential for public good provisioning has not been studied for host glycans. In this study, we therefore aimed to examine the degradation abilities of CS and HA by a diverse panel of intestinal *Bacteroides* species in order to extend current knowledge on the CS-HA PUL as well as evaluate the extent of potential public good provisioning and utilization due to degradation of these two host-derived glycans among the *Bacteroides*.

## Materials and methods

### Carbohydrates

Chondroitin sulfate A sodium salt (CS) from bovine trachea, hyaluronic acid sodium salt (HA) from *Streptococcus equi* and D-glucuronic Acid (GlcA) were obtained from Merck (Germany). Glucose, N-acetyl-D-galactosamine (GalNAc) and N-acetyl-D-glucosamine (GlcNAc) were obtained from Carl Roth (Germany).

### Culturing of *Bacteroides* strains

In total, 17 different *Bacteroides* species (Supplementary Table S1) were cultured under anaerobic conditions (atmosphere: 85% N2, 10% CO2, 5% H2, Coy Laboratory Products, United States) at 37°C using M9 minimal media without glucose (unless stated otherwise, see Supplementary Table S2; Neidhardt et al., 1974) supplemented with 0.05 g cysteine, 1 mL 0.5% hemin, 0.5 mL 0.5% vitamin K1, 0.1 mL 2% FeSO4 and 0.05 mL 0.01% vitamin B12 per liter M9. Either 0.5% CS, HA, or glucose was added as a carbon source. All cultures were pre-grown overnight on M9-glucose before being switched to alternative carbon sources. Cultures were then grown for at least 16 h in a plate reader (Multiscan Go, Thermo Fischer Scientific, Germany) inside the anaerobic chamber. Growth was measured in the plate reader *via* optical density (OD_600_) every 30 min with 10 s shaking before measurement. Cultures were grown in triplicate per run and at least two biological replicates were performed. Cultures that did not grow on the positive control (glucose) were excluded as values for the positive control were used for normalization. For each strain, growth rate, generation time, and maximum optical density was calculated in R (R Core Team, 2021) using the Growthcurver package (Sprouffske, 2020). Raw data was blanked by subtracting the starting OD, and all data was trimmed to 19 h as stationary phase was reached. For growth on conditioned media, values were normalized to the maximum OD_600_ value obtained on glucose for comparison between conditions and strains. Graphs were visualized using the R package Pheatmap (Kolde, 2019).

### Conditioned media

Conditioned media (CM) was prepared by growing bacteria until late log phase, after which cultures were harvested, spun down at 13.4 rpm for 10 min, and the supernatant was filtered twice using a 0.2 μm poresize. Conditioned media was diluted 1:1 with fresh M9 medium without an additional carbon source, after which cultures were inoculated and introduced into the plate reader. All CM was incubated at 37°C and measured as negative control to prevent contamination from the donor strain.

### Fluorophore-assisted carbohydrate electrophoresis

To determine if any sugars were left in the CM, a fluorophore-assisted carbohydrate electrophoresis (FACE) was performed according to (Midura et al., 2018). Briefly, CM was vacuum-dried and then incubated with 5 μL 2-Amino-9(10H)-acridinone (AMAC) solution at 37°C for 18 h. FACE gels were prepared using 25 μL 10% ammonium persulfate and 5 μL TEMED per 5 mL of acrylamide solution. Gels were loaded with 3 μL AMAC-labelled CM and run for 50 min at 300 V and 60 mA in a chamber with chilled TBE.

### Transcriptomic analysis of *Bacteroides thetaiotaomicron*

RNA was extracted from B. theta cell pellets grown on either CS, HA or a mixture containing GalNAc, GlcNAc, and GlcA (harvested from 30 mL late log phase cultures) using the innuPREP RNA Mini Kit 2.0 (Analytik Jena) following the manufacturer’s protocol. Residual DNA in the total RNA extracts was digested using TURBO DNAse (Thermo Fisher Scientific) following the manufacturer’s instructions. Absence of residual DNA contamination was confirmed by 16S rRNA gene targeted PCR (using primers 515F^1^ and 806R^2^, following the amplification protocol published in Pjevac et al., 2021). The riboZero Plus Kit (Illumina) was used to deplete ribosomal RNA (rRNA) from the total RNA extracts, following the manufacturer’s protocol. Single-index barcoded sequencing libraries were prepared from rRNA-depleted RNA using the NEBNext® Ultra™ II Directional RNA Library Prep Kit for Illumina (NewEngland Biolabs) following the manufacturer’s protocol. Sequencing libraries were then pooled and sequenced on a HiSeq3000 (Illumina) in paired-end mode (150 cycles, 2× 75 bp reads) at the Biomedical Sequencing Facility (BSF) of the CeMM Research Center for Molecular Medicine of the Austrian Academy of Sciences/Joint Microbiome Facility (JMF) of the Medical University of Vienna and the University of Vienna (project ID JMF-2012-5).

Reads were quality filtered and trimmed with bbduk (ref = adapters. Ktrim = r, k = 23, mink = 11, hammingdistance = 1, qtrim = r, trimq = 28, minavgquality = 15, minlength = 50) and mapped to the *B*. *theta* reference genome (Xu et al., 2003) using bbmap (minratio = 0.96324, pairedonly = t, ambiguous = toss/best/all, Bushnell, n.d.). Mapped read pairs were counted using featureCounts (−s 2; Liao et al., 2014). One library failed and was removed from further analysis. Next, DESeq was used to analyze count data using the standard pipeline with alpha = 0.05 (Love et al., 2014). Genes were compared with CS against the mix, HA against mix and CS against HA. Genes with an adjusted value of *p* <0.05 and a log-2 fold-change smaller than-2 or greater than 2 were considered to be significantly down or up regulated. Vegan was used for permutational multivariate analysis of variance (PERMANOVA; Wagner et al., 2020).

### Comparative genomics

Orthofinder, using default settings, was used to compare relevant CS-HA degradation PULs from *B*. *theta* with the 17 other *Bacteroides* species used for CS-HA growth analysis (Emms and Kelly, 2019). Degradation abilities were assigned based on growth results on CS and HA, and those orthogroups only present in CS-only and/or CS-HA degraders were filtered out. Next, 53 *Bacteroides* genomes (translated CDS) were downloaded from NCBI and BlastP was used to blast specific genes from the *B*. *theta* PULs against these genomes to create a gene presence/absence table (cut-off evalue = 1e^−40^). To create a phylogenetic tree, 43 markers were aligned and concatenated using default settings in CheckM for the lineage workflow (Parks et al., 2015) after which a model was estimated with modelFinder (Kalyaanamoorthy et al., 2017). Using Bayesian information criterion scores, the best-fit model was LG + F + R3. Next a tree was calculated using default settings in IQ-TREE 2 (Minh et al., 2020) with the addition of ultrafast bootstraps (Hoang et al., 2018). For visualization, the tree was then uploaded to iTOL (Letunic and Bork, 2021) and combined with the gene presence/absence data.

## Results

### Not all chondroitin sulfate degraders can degrade hyaluronan

Until recently, it was believed that all *Bacteroides* capable of degrading CS were also capable of degrading HA (Salyers et al., 1977; Ndeh et al., 2020). To evaluate this, we grew 17 *Bacteroides* species on a minimal media containing either 0.5% CS or HA (Figure 1B). Literature states structures of CS and HA are similar despite the fact that CS is heavily sulfated and they have different monosaccharide compositions as stated previously (Bartlett and Park, 2010). We initially hypothesized that HA degradation capability will be more widespread, and only strains encoding the requisite sulfatases will be able to degrade CS. In total, seven species were able to grow on both CS and HA (*B*. *acidifaciens*, *B*. *caccae*, *B*. *clarus*, *B*. *finegoldii*, *B*. *ovatus*, *B*. *thetaiotaomicron*, and *B*. *xylanisolvens*; Figure 1B). Five strains grew on CS but not on HA (*B*. *cellulosilyticus*, *B*. *eggerthii*, *B*. *intestinalis*, *B*. *oleiciplenus*, and *B*. *stercoris*). Additionally, five strains could not degrade either compound (*B*. *dorei*, *B*. *fluxus*, *B*. *fragilis*, *B*. *uniformis*, and *B*. *vulgatus*). Strains were also tested for the ability to grow on the monosaccharide subunits of CS (GalNAc) and HA (GlcNAc), and most species were able to grow on both (Figure 1B), with the exception of *B*. *clarus* and *B*. *cellulosilyticus*, which were not able to grow on GalNAc, and *B*. *oleiciplenus*, which was not able to grow on GlcNAc. For each strain, the growth rate (Figure 1C) was calculated. There were differences between individual strains but no clear distinction between degradation groups.

### Largely overlapping transcriptional regulation of chondroitin sulfate and hyaluronan utilization by *Bacteroides thetaiotaomicron*

**Figure 2:**
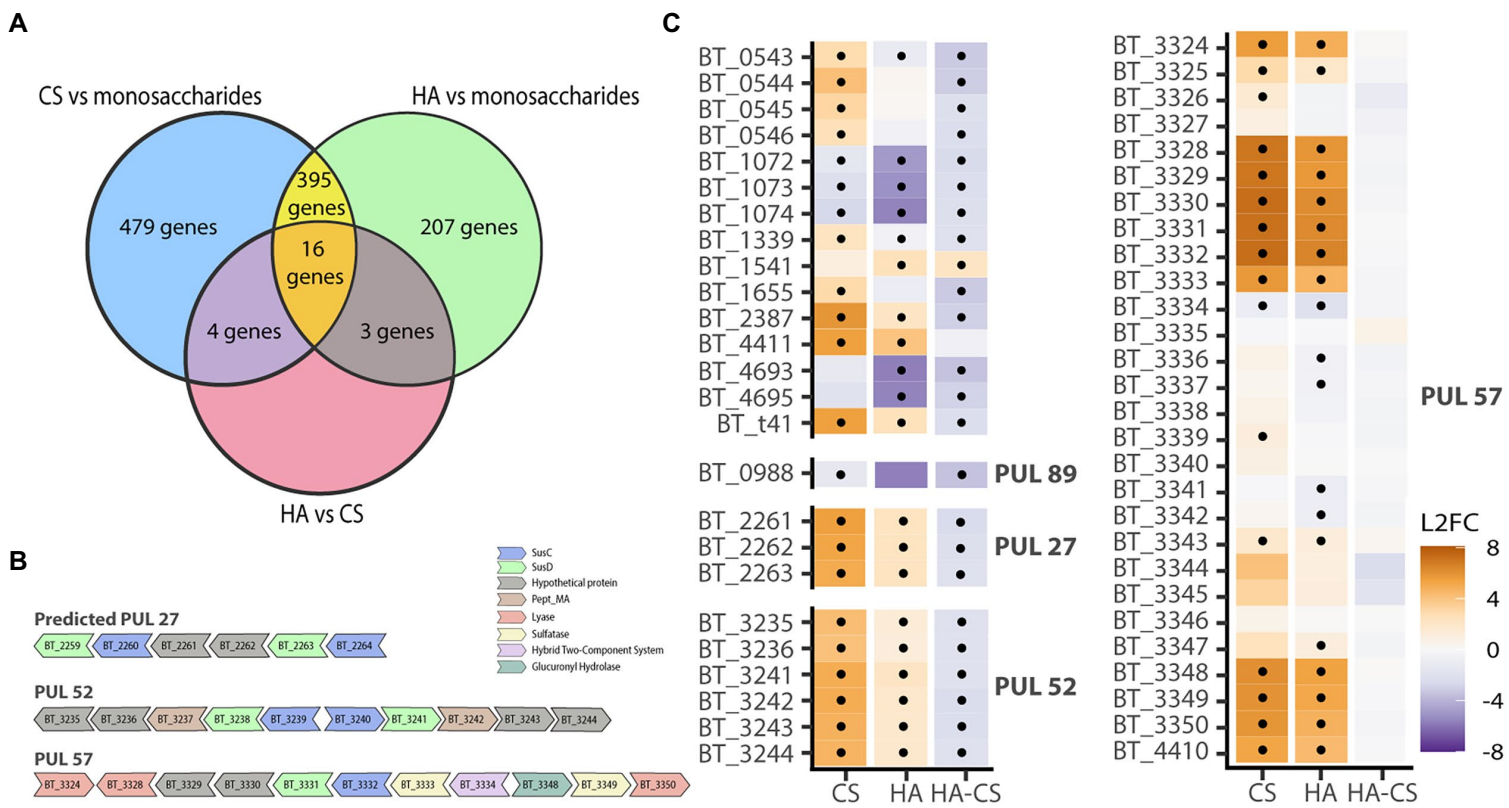
Transcriptomics does not indicate a separate pathway for HA degradation. *B*. *theta* was grown on either CS, HA or a control mix containing GalNac, GlcNac or GlcA. **(A)** Venn diagram showing overlapping differentially expressed genes in the different growth conditions. **(B)** Polysaccharide Utilization Loci (PUL) containing differentially expressed genes for CS and/or HA. **(C)** Heatmap showing log 2 fold change (L2FC) for CS – mix, HA-mix or HA compared with CS. Genes are grouped by PULs. PUL57 is the known PUL for CS & HA degradation and does not differ for CS or HA. Dots indicate statistically significant genes (*p* < 0.05 and L2FC of either ≥2 or ≤ −2).

*Bacteroides theta* is one of the best characterized gut commensal bacteria and is a widely used model organism. To evaluate if *B*. *theta* has separate pathways for CS and HA degradation, it was grown on either CS or HA and subjected to RNA sequencing. As an additional control, *B*. *theta* was grown on a mixture of GalNAc, GlcNAc, and GlcA. On average, 4,663 genes were expressed per library (Supplementary Table S3). Nutrient source explained 69% of the total variation in gene expression profiles (PERMANOVA; *p* = 0.002). When compared to growth on the simple sugar mixture control, there were 802 genes upregulated and 88 downregulated during growth on CS (padj ≤0.05 and ≥ 2 log2 fold change; Supplementary Table S4), and 399 genes upregulated and 219 downregulated during growth on HA. When directly comparing gene expression profiles during growth on CS or HA, there were 23 genes downregulated on HA and 1 gene upregulated (Supplementary Table S4). For CS degradation there was one PUL with unknown function which showed consistently higher transcription across all genes on CS (PUL 52; Figure 2; Supplementary Table S5). This PUL runs from BT_3235 to BT_3244 and consists of hypothetical proteins and two sus-D homologs. This PUL does not seem to encode known sulfatases, meaning that all sulfatases required for the degradation of CS are found in the characterized PUL for CS and HA degradation, PUL 57. This PUL ranges from BT_3324 to BT_3350 with the addition of BT_4410 being co-regulated by the glycoside hydrolase (BT_3334) in the PUL. Genes previously reported in relation to degradation of CS and HA were manually verified yet had differential expressions below a two log2-fold-change or were found not to have a significant value of p. Our results do not indicate a difference in expression between CS or HA for any of the genes in PUL 57 (Figure 2), thus they do not suggest that there is a separate pathway in *B*. *theta* for HA degradation. However, the results do show upregulation of PUL 57 when comparing either of the GAGs with simple sugars, validating the transcriptomic approach.

### Chondroitin sulfate and hyaluronan degradation ability is reflected in the gene repertoire of gut *Bacteroides*

As transcriptomic results did not suggest a separate pathway for HA degradation, we suspected there may be specific genes necessary for HA degradation that were lost in CS-only degraders. First, we compared gene orthologs of *B*. *theta* to the 17 *Bacteroides* strains used for physiological experiments. In total, 1,193 orthogroups were assigned using 93.2% of the genes. Using the orthogroup gene counts table, we selected only those orthogroups which contained genes for CS and/or CS-HA degraders yet did not contain genes from non-degraders. This resulted in four orthogroups; one for CS-only degraders (OG0003539), one for all degraders (OG0002347) and two for CS-HA degraders (OG0002608 and OG0003140; Supplementary Table S6). The orthogroup for all degraders contained the known CS lyase, BT_3350 (Martens et al., 2008). The two orthogroups containing the other known CS-lyases (BT_3324 in OG0002222 and BT_4410 in OG0002357; Raghavan et al., 2014) contained genes from the non-degrader *B*. *uniformis*. For the CS-HA degraders the two orthogroups contained the hypothetical protein BT_4411, and the hypothetical protein BT_3328.

**Figure 3:**
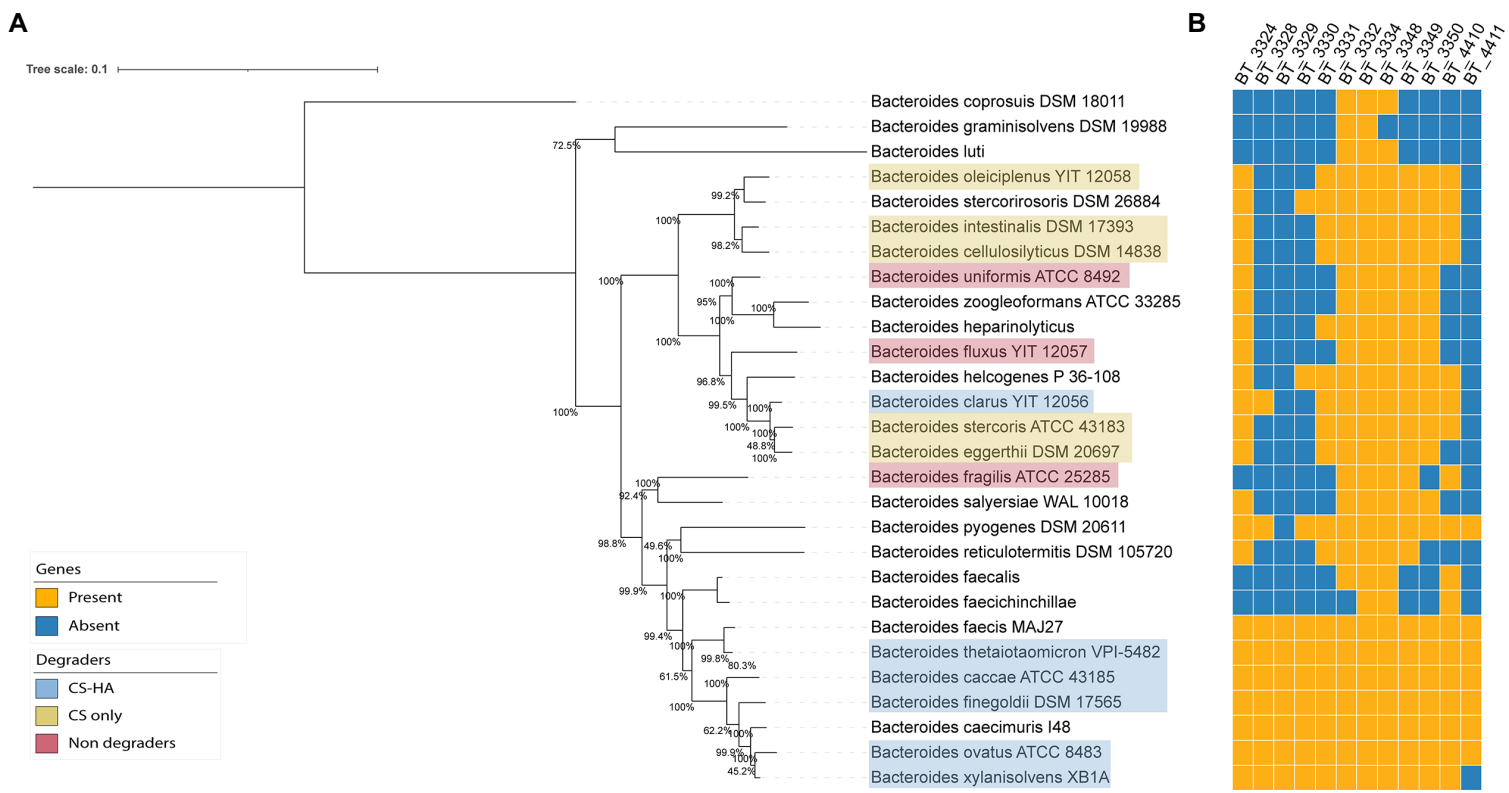
**(A.B)** Phylogenomic tree of gut *Bacteroides* and gene repertoire of putative CS and HA utilization genes. Phylogenetic tree of all *Bacteroides* species from NCBI with a translated CDS file. Genomes were aligned with CheckM (Parks et al., 2015) and the tree was calculated using iqtree2 (Minh et al., 2020). Clustering is not based on degradation capabilities of CS and/or HA. BlastP (cut-off evalue = 1e^−40^) was used to create a presence/absence table of genes from *B. theta* (potentially) required for chondroitin sulfate (CS) and hyaluronan (HA) degradation based on literature and transcriptomic results. BT_3328 seems to be the crucial gene for HA degradation as bacteria without this gene are incapable of growth on HA.

Next, a phylogenomic tree of 53 gut *Bacteroides* strains was created to determine if there was phylogenetic conservation of CS and HA degradation ability (Figure 3A). We did not observe a phylogenetic separation between degraders and non-degraders, nor between CS and CS-HA degraders. CS degraders were spread over numerous clades. Although most CS-HA degraders cluster together, *B*. *clarus* prevents CS-HA degraders from being a monophyletic taxon, which could suggest that the CS-only phenotype occurs due to lineage-specific gene loss.

We then wanted to evaluate the conservation of the previously-identified *B*. *theta* genes of interest among the gut *Bacteroides* and thus used BlastP to identify the gene content of other *Bacteroides* species (Figure 3B). An incomplete cluster of *Bacteroides* strains containing the lyase BT_3328 was present, however *B*. *pyogenes* and *B*. *clarus* were in separate clades. Predicted PUL 27 and PUL 52, which were upregulated in *B*. *theta* when grown on CS, were also included in the Blast search (Supplementary Table S5). For PUL 27, the genes were only found in 2–3 other *Bacteroides* species. For PUL 52 there was a wider distribution across the different species, although there was no clear distinction between CS and/or HA degraders in comparison to non-degraders. Although there was not a distinguished CS-HA degrader clade, all tested HA-degrading strains had the BT_3328 lyase, whereas this was absent in all non-HA degrading strains.

### Breakdown products of chondroitin sulfate and hyaluronan differ between species

**Figure 4:**
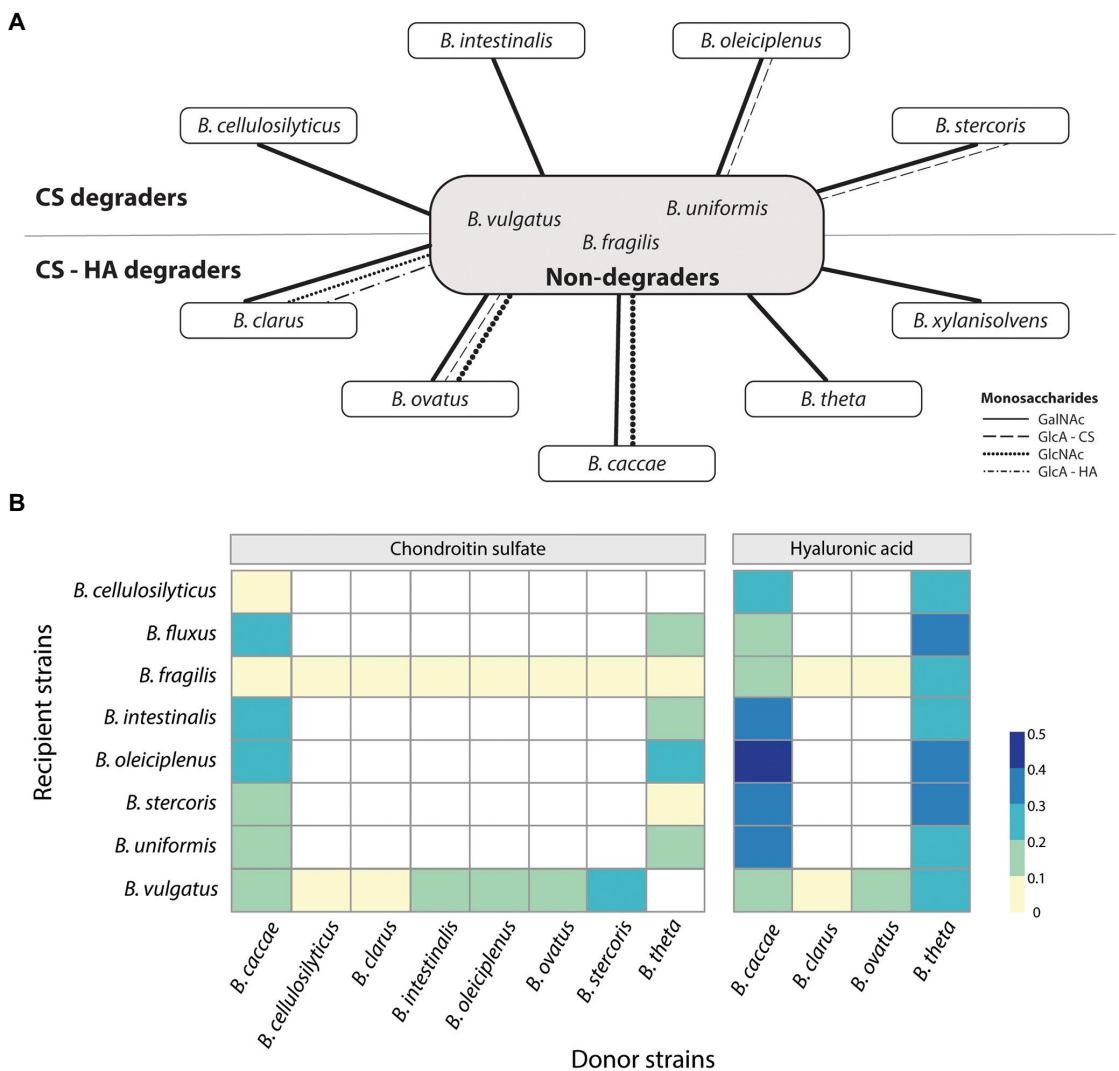
Sharing of public goods depends on carbon source and donor and recipient strains. **(A)** Theoretical public goods provision by CS and CS-HA degraders to non-degraders based on fluorophore-assisted carbohydrate electrophoresis results. CS, chondroitin sulfate; HA, hyaluronan; GalNAc, N-Acetylgalactosamine; GlcNAc, N-Acetylglucosamine; GlcA – CS, glucuronic acid from CS; GlcA – HA, glucuronic acid from HA. **(B)** Not all donor strains (selected CS-HA and CS-only degraders) can supply public goods. Similarly, not all non-degraders are capable of growth on CM. HA donors supply better growth than CS donors. Donor strains were grown until late exponential after which the supernatant was filtered and diluted 1:1 with fresh M9 medium. Recipient strains were then grown on this Conditioned Media (CM) for 20 h. Optical densities (OD) were normalized to the maximum value grown on glucose. OD at 19 h is shown. White boxes indicate N/A values.

We next wanted to evaluate if the breakdown products from CS and/or HA degradation are potentially available as public goods available for other *Bacteroides*. To do so, fluorophore-assisted carbohydrate electrophoresis (FACE) was used to stain spent supernatant. For all strains tested, CS degradation showed numerous different break-down products, including a strong signal for GalNAc and a variety of di-and oligosaccharides at varying signal strength (Figure 4A; Supplementary Figure S1 for a representative image). For HA, *B*. *caccae* and *B*. *ovatus* showed a strong signal for GlcNAc, no signal for GlcA, but a third band of an unknown breakdown product (Supplementary Figure S1; Supplementary Table S7). For *B*. *ovatus*, *B*. *stercoris*, and *B*. *xylanisolvens* media was harvested at intervals for 24 h to follow the degradation of CS (Supplementary Figure S2 for a representative image). For all three there was one band observed already after 2 h, despite no growth being observable yet *via* OD. After 24 h some bands were lost or were less intense, yet degradation products remained in the medium. *B*. *ovatus* was also grown on HA and showed only two bands during the entire timespan with the first, unknown band occurring after 2 h and the GlcNAc band appearing after 4 h. These unknown bands are presumably intermediate break down products of CS and HA in the form of di-and/or oligosaccharides. Based on FACE results, it seems that all strains produce extracellular sugars outside of the periplasm which could potentially be used by other (non-degrading) bacteria and used as public goods (Figure 4A). To test this, degraders were grown on either CS or HA until late exponential phase, supernatant was spun down and filtered to remove bacteria and is now conditioned media (CM). Next, we diluted the CM with fresh M9 medium without an additional carbon source and inoculated non-degrading bacteria or CS-only degraders and measured OD. Interestingly, CM from donors grown on HA provided more non-degrader growth then CM from CS (Figure 4B). Additionally, some non-degraders were better at growing on CM than others. *B*. *fragilis* for example, was incapable of growing on any of the CMs from CS, regardless of donor whereas *B*. *oleiciplenus* showed good growth. Interestingly enough, the growth results and CM visualization do not align. FACE-PAGE for *B*. *theta* only indicated degradation products for CS yet and very limited for HA, yet non-degraders were capable on growing on CM from HA. In contrast, *B*. *clarus* showed a multitude of degradation products with FACE-PAGE, yet non-degraders were incapable of growth on *B*. *clarus* CM.

## Discussion

It has been thought that bacteria capable of degrading CS were also able to degrade HA, as the characterized lyases worked on both polysaccharides (Salyers et al., 1977; Ndeh et al., 2020). We therefore expected that all tested strains with homologs of these lyases would be able to degrade HA, and those which additionally encoded sulfatases would be able to degrade both CS and HA. Our results do show that there is a separate group of *Bacteroides* only capable of degrading CS (Figure 1A). These results corroborate previous results from Ndeh et al. (2020) and show that this holds true for a larger subset of *Bacteroides*. They did not, however, explore the broader significance of these findings for HA metabolism (Ndeh et al., 2020). In this study we set out to investigate the possibility of public goods for host-derived polysaccharides, but upon the finding of the CS-only degrader groups additionally wanted to deduce a potential mechanism for HA degradation.

Using the model organism *B*. *theta*, we concluded that HA degradation did not require a separate pathway from CS breakdown (Figure 2). This is in line with previous gene expression microarray results from Martens and co-workers (Martens et al., 2008, 2011). Our phylogenetic analysis of *Bacteroides* strains tested suggests the loss of a gene required for HA utilization in CS-only degraders since (with the exception of *B*. *clarus*) CS-HA degraders cluster together (Figure 3A). Comparative genomic analyses presented in this study indicated that the lyase BT_3328 and hypothetical protein BT_4411 may be together necessary for HA degradation (Figure 3B). BT_4411 has been predicted to be in an operon together with the known HA lyase BT_4410 and is similar to carbohydrate binding domain PF02018, theythus may function together to degrade HA (Liu et al., 2021). Previous knockouts of both BT_4410 and BT_4411 show growth deficits on HA (Ndeh et al., 2020; Liu et al., 2021). However, our results indicate that BT_4411 is absent in two of the CS-HA degraders, we propose this could be a second pathway pathway for HA degradation, similar to the pectin pathway where *B*. *theta* also has multiple susC-D transporter pairs (Luis et al., 2018).

The CS/HA lyase BT_3328 has been previously identified as the first cell surface lyase for *B*. *theta* (Ndeh et al., 2018) and interacts together with the predicted surface glycan binding proteins (SGBP) BT_3329 and BT_3330 at the outer membrane. The BT_3328 lyase has been shown to prefer larger molecules with a high degree of polymerization (Ndeh et al., 2018). Since the other lyases capable of HA degradation are all found within the periplasm, HA may need to be partially broken down outside of the cell by BT_3328 before it can be imported. Previous knockouts for BT_3328 are inconclusive, with one study showing no growth deficits for 1% CS or HA (Ndeh et al., 2018), one study showing a modest growth defect on 0.5% HA and another showing growth on HA to be strongly decreased (Liu et al., 2021). The latter study also shows knockouts for the SGBPs BT_3329 and BT_3330 negatively affecting growth on HA (Liu et al., 2021). Interestingly, *B*. *clarus* does not have BT_3329 nor BT_3330 yet it may have similar SGBPs which can bind HA next to the BT_3328 lyase.

**Figure 5:**
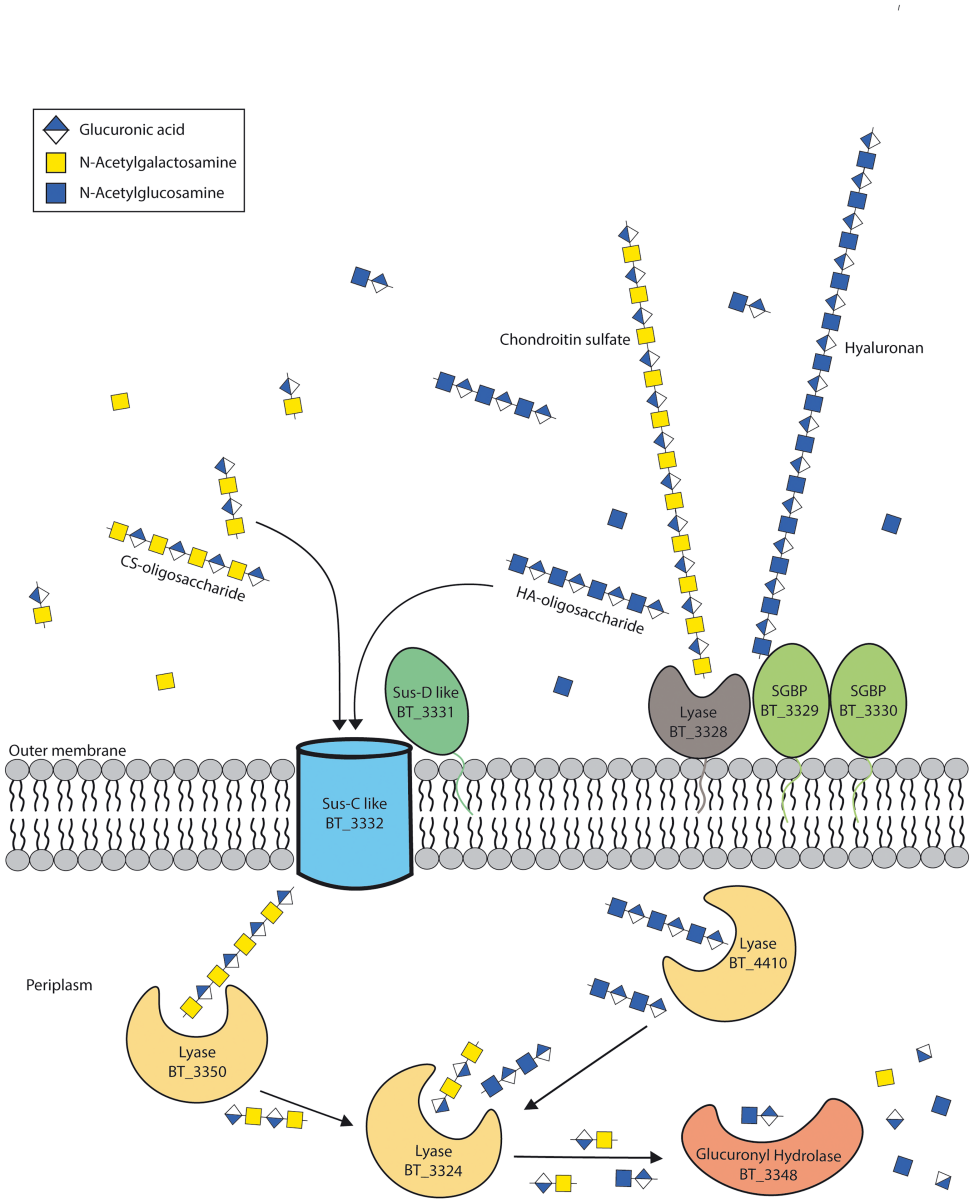
Suggested model for hyaluronan breakdown. Based on our results, we propose that the lyase BT_3328 and surface glycan binding proteins (SGBP) BT_3329 and BT_3330 are required to cleave hyaluronan (HA) before transport and further degradation in the periplasm. Adapted from Ndeh et al. (2020).

Molecular weights for both CS and HA are highly variable, for CS they range mostly between 50–100 kDa (Kwan Tat et al., 2010), whereas HA is much larger with a range between 1,000–8,000 kDa (Cowman et al., 2015). A recent paper studying the SusC/D complex for levan degradation have shown there is an upper limit in size for import through this complex (Gray et al., 2021). They suggest 5 kDa to be the upper limit for saccharide import. We thus propose that HA is too large to be transported into the cell with the SusC-SusD transporters of the CS-HA PUL 57. HA would thus require the SGBPs BT_3329 and BT_3330 to bind HA followed by the lyase BT_3328 creating HA oligosaccharides after which it can be imported and further degraded by periplasmic CS-HA lyases (Figure 5). This hypothesis is supported by the lack of BT_3328-BT_3330 in CS-only degraders in our bioinformatic analysis, yet would need wet-lab confirmation by, e.g., double and triple mutants and/or knock-ins of these genes into CS-only degraders.

FACE analysis presented in this study showed that *Bacteroides* strains produce extracellular breakdown products from CS and HA degradation, suggesting the potential for providing public goods to other bacteria (Figure 4A). For CS, this was initially surprising as the lyases are inside the periplasm, however, following the results from Gray et al., CS would be above the maximum threshold size of direct import by SusC/D and would thus also require breakdown by an extra-cellular lyase such as BT_3328 (Gray et al., 2021). Additionally, Koropatkin et al., suggest there is also a minimal size limit to the SusC/D system with monosaccharides being imported *via* a non-Sus system (Koropatkin et al., 2008). Ndeh et al. (2020) reported that the smallest unit of CS and HA degradation by the lyase BT_3328 are disaccharides, so there may be an additional lyase which can then cleave disaccharides to monosaccharides as is seen for the SusC/D system for pectin (Li et al., 2021). In this study, when looking at CS degradation over time it was observed that bands corresponding to GlcA were present in the media but disappeared after 9 h, suggesting this was preferred over GalNAc as a carbon source. This is surprising as GalNAc and GlcNAc are cytotoxic for epithelial cells and need to quickly be removed from the lumen (Lee et al., 2009), however both GalNAc and GlcNAc require additional kinases for phosphorylation before they can be metabolized (Ndeh et al., 2020). Lastly, it seems that even when monosaccharides are present in the CM, not all recipient strains are capable of growth (Figure 4B). It should be noted that concentrations of sugars were not quantified or standardized before CM inoculation, resulting in differing concentrations between CM samples, and therefore it is not possible to distinguish between the ability to grow on breakdown products and the availability of breakdown products in different CMs Nevertheless, plausible explanations for the difference of growth capabilities on CM include the presence of inhibitory compounds like antimicrobials that are packaged in outer membrane vesicles and secreted extracellularly (Coyne et al., 2019). Another possible explanation for selective cross-feeding could be the degree of polymerization of break-down products with not all species being capable of degrading the higher degree components or lacking pathways and deaminases to use monosaccharides (Luis et al., 2018). Thus, our results show *Bacteroides* species not only have differing abilities of GAG degradation and public goods distribution but could also have preference to whom they provide nutrients.

In conclusion, although CS and HA degradation have been extensively studied in *B*. *theta*, not much is known about the degradation in other *Bacteroides* species. We have shown that although multiple *Bacteroides* species encode periplasmic lyases which should be able to break down both CS and HA, some cannot utilize HA. We suggest this is due to the loss of the outer membrane lyase BT_3328 and SGBPs BT_3329 and BT_3330. However, due to cooperative degradation and public good provisioning, loss of these genes may not put these species at a selective disadvantage in the mucosal ecosystem.

## Data availability statement

The datasets presented in this study can be found in online repositories. The names of the repository/repositories and accession number (s) can be found at: https://www.ncbi.nlm.nih.gov/sra, PRJNA837438.

## Author contributions

AO, FP, and DB designed the experiments. AO performed all lab work assisted by GN, with the exception of sequencing which was done by the Joint Microbiome Facility. AO, BH, CH, and DB performed bioinformatic analyses. All authors read and approved the manuscript.

## Funding

This project was funded by the Austrian Science Fund (FWF; P27831-B28) and European Research Council (Starting Grant: FunKeyGut 741623). Work in the laboratory of the Centre for Microbiology and Environmental Systems Science was supported by Austrian Science Fund project MAINTAIN DOC 69 doc.fund.

## Conflict of interest

The authors declare that the research was conducted without any commercial or financial relationships that could be construed as a potential conflict of interest.

## Publisher’s note

All claims expressed in this article are solely those of the authors and do not necessarily represent those of their affiliated organizations, or those of the publisher, the editors and the reviewers. Any product that may be evaluated in this article, or claim that may be made by its manufacturer, is not guaranteed or endorsed by the publisher.

## Footnotes

^1^ https://www.ncbi.nlm.nih.gov/pubmed/26271760

^2^ https://doi.org/10.3354/ame01753
