# Supplementary material for: Nutrient niche specificity for glycosaminoglycans is reflected in polysaccharide utilization locus architecture of gut Bacteroides species
Source: Front Microbiol. 2022 Nov 29;13:1033355. doi: 10.3389/fmicb.2022.1033355 (PMC9745678; doi:10.3389/fmicb.2022.1033355)
Supplement: Supplementary file 1 [file Data_Sheet_1.PDF]

Supplementary Material

Supplementary table 1: Strains used in this study

Organism	Strain	Accession
Bacteroides acidifaciens	DSMZ15896 (JCM 10556)	
Bacteroides caccae	Bacteroides caccae 19024, type strain (ATCC 43185)	GCA_000169015.1_ASM16901v1_translated_cds.faa
Bacteroides cellulosilyticus	Bacteroides cellulosilyticus CRE21 DSM No.: 14838, Type strain	GCA_000158035.1_ASM15803v1_translated_cds.faa
Bacteroides clarus	Bacteroides clarus DSM No.: 22519, Type strain (YIT 12056)	GCA_000195615.1_ASM19561v1_translated_cds.faa
Bacteroides dorei	Bacteroides dorei DSM No.: 17855, Type strain	
Bacteroides eggerthi	Bacteroides eggerthii DSM No.: 20697, Type strain	GCA_000155815.1_ASM15581v1_translated_cds.faa
Bacteroides finegoldii	Bacteroides finegoldii DSM No.: 17565, Type strain	GCA_000156195.1_ASM15619v1_translated_cds.faa
Bacteroides fluxus	Bacteroides fluxus DSM No.: 22534, Type strain (YIT 12057)	GCA_000195635.1_ASM19563v1_translated_cds.faa
Bacteroides fragilis	Bacteroides fragilis 2151, type strain (ATCC 25285)	GCA_001997325.1_ASM199732v1_translated_cds.faa
Bacteroides intestinalis	Bacteroides intestinalis DSM No.: 17393, Type strain	GCA_000172175.1_ASM17217v1_translated_cds.faa
Bacteroides oleiciplenus	Bacteroides oleiciplenus DSM NO.: 22535, Type strain (JCM 16102 / YIT 12058)	GCA_000315485.1_Bact_olei_YIT_12058_V1_translated_cds.faa
Bacteroides ovatus	Bacteroides ovatus 1896, type strain	GCA_002959635.1_ASM295963v1_translated_cds.faa
Bacteroides stercoris	Bacteroides stercoris DSM No.: 19555, Type strain	GCA_900106605.1_IMG-taxon_2693429855_annotated_assembly_translated_cds.faa
Bacteorides thetaiotaomicron	Bacteroides thetaiotaomicron 2079, type strain	GCA_000011065.1_ASM1106v1_translated_cds.faa

Bacteroides uniformis	Bacteroides uniformis 6597, type strain (ATCC8492)	GCA_000154205.1_ASM15420v1_translated_cds.faa
Bacteroides vulgatus	Bacteroides vulgatus 1447, type strain (ATCC8482)	
Bacteroides xylanisolvens	Bacteroides xylanisolvens DSM No.:18836, Type strain XB1A	GCA_000210075.1_ASM21007v1_translated_cds.faa

Supplementary table 2: Components of M9 minimal media, adapted from Neidhardt et al., 1974

	**1 L**
NH_4Cl	1g
Na_2HPO_4	6g
KH_2PO_4	3g
NaCl	0.5g
1M $CaCl_2.2H_2O$	100 µl
1M $MgSO_4.7H_2O$	1 ml
5% Cysteine	10 ml
0.5% Hemin	1 ml
0.5% $VitK_1$	500 µl
2% $FeSO_4$	100 µl
0.01% Vit B_{12}	50 µl

Supplementary table 3: Library results per sample

	**Reads**	**Expressed genes**
BT CS 1	180096834	4891
BT CS 2	14230812	3619
BT CS 3	263558	4616
BT CS 4	350	
BT HA 1	25749471	4829
BT HA 2	7121958	4730
BT HA 3	36713662	4856
BT HA 4	2125904	4480

BT mix 1	16542308	4769
BT mix 2	36725544	4812
BT mix 3	36179577	4818
BT mix 4	96901885	4873

Supplementary table 4: Number of statistically significant genes

	**padj <= 0.05**	**L2FC >= 2**	**L2FC <= -2**
CS	1659	802	88
HA	2825	399	219
HA vs CS	42	1	23

Supplementary table 5: *B. theta* genes upregulated when comparing CS to HA

**PUL**	**Gene**	**Function**	**CSL2FC**	**CS padj**	**HAL2FC**	**HA padj**	**HACSL2FC**	**padj**
CS upregulated								
	BT_0543	Glutamine synthetase	2.8	0.00	-1.2	0.00	-3.2	0.00
	BT_0544	Ammonium transporter	4.1	0.00	0.3	0.46	-3.1	0.00
	BT_0545	Nitrogen regulatory protein P-II	3.1	0.00	0.2	0.63	-2.2	0.02
	BT_0546	Uncharacterized protein	2.5	0.00	-0.6	0.09	-2.3	0.00
**PUL 89**	BT_0988	Magnesium-transporting ATPase, P-type 1 (EC 7,2,2,14) (Mg(2+) transport ATPase, P-type 1)	-1.5	0.17	-5.7	0.00	-3.5	0.03
	BT_1072	Uncharacterized protein	-1.6	0.00	-4.9	0.00	-2.6	0.00
	BT_1073	DUF4136 domain-containing protein	-2.2	0.00	-5.2	0.00	-2.3	0.00
	BT_1074	OMP_b-brl domain-containing protein	-2.7	0.00	-5.6	0.00	-2.2	0.03
	BT_1339	Undecaprenyl-phosphate alpha-N-acetylglucosaminyltransferase	2.2	0.00	-0.6	0.00	-2.1	0.03
	BT_1655	Uncharacterized protein	2.9	0.00	-1.0	0.06	-3.2	0.00
	BT_2261	Uncharacterized protein	5.4	0.00	2.2	0.00	-2.5	0.00

**Predicted PUL 27**	BT_2262	Uncharacterized protein	5.2	0.00	2.2	0.00	-2.2	0.01
	BT_2263	Putative lipoprotein	5.0	0.00	2.2	0.00	-2.1	0.00
	BT_2387	O-acetylhomoserine (Thiol)-lyase	5.8	0.00	2.1	0.00	-3.0	0.00
**PUL 52**	BT_3235	Uncharacterized protein	4.2	0.00	1.3	0.00	-2.1	0.01
	BT_3236	Uncharacterized protein	4.0	0.00	1.2	0.00	-2.0	0.01
	BT_3241	SusD homolog	4.9	0.00	2.1	0.00	-2.0	0.01
	BT_3242	Uncharacterized protein	4.9	0.00	1.8	0.00	-2.4	0.00
	BT_3243	DUF4987 domain-containing protein	4.7	0.00	1.7	0.00	-2.3	0.01
	BT_3244	BACON domain-containing protein	4.6	0.00	1.7	0.00	-2.1	0.01
	BT_4693	Cation efflux system protein	-1.4	0.18	-5.7	0.00	-3.5	0.01
	BT_4695	Outer membrane efflux protein	-1.9	0.06	-5.6	0.00	-2.9	0.00
	BT_t41	NA	5.4	0.00	2.4	0.00	-2.3	0.04
HA upregulated								
	BT_1541	Putative transmembrane protein	1.0	0.06	2.4	0.00	2.1	0.00

Supplementary table 6: Comparative genomics, relevant orthogroup gene counts ordered by degradation ability

**Genome**	**Organism**	**Degrader**	**OG0002222**	**OG0002347**	**OG0002357**	**OG0002608**	**OG0003140**	**OG0003539**
GCA_000154845.1_ASM15484v1_protein	B. eggerthii	CS	1	1	1	0	0	1
GCA_000155815.1_ASM15581v1_protein	B. cellulosilyticus	CS	1	1	1	0	0	1
GCA_000965785.1_ASM96578v1_protein	B. intestinalis	CS	1	1	1	0	0	1
GCA_001314995.1_ASM131499v1_protein	B. oleiciplenus	CS	1	1	1	0	0	1
GCA_001318345.1_ASM131834v1_protein	B. stercoris	CS	1	1	1	0	0	1
GCA_001404475.1_13414_6_24_protein	B. ovatus	CSHA	1	1	1	1	1	0
GCA_001412315.1_ASM141231v1_protein	B. finegoldii	CSHA	1	1	1	2	1	0

GCA_001578555.1_ASM157855v1_protein	B. plebeius	CSHA	1	1	1	2	1	0
GCA_001578635.1_ASM157863v1_protein	B. clarus	CSHA	1	1	1	2	1	0
GCA_001915515.1_ASM191551v1_protein	B. xylanisolvens	CSHA	1	1	1	1	1	0
GCA_001915535.1_ASM191553v1_protein	B. caccae	CSHA	1	1	1	1	1	0
GCA_002160225.1_ASM216022v1_protein	B. theta	CSHA	1	1	1	1	1	0
GCA_002160605.1_ASM216060v1_protein	B. fragilis	Non	0	0	0	0	0	0
GCA_002161115.1_ASM216111v1_protein	B. vulgatus	Non	0	0	0	0	0	0
GCA_002222615.2_ASM222261v2_protein	B. uniformis	Non	1	0	1	0	0	0
GCA_900106605.1_IMG-taxon_2693429855_annotated_assembly_protein	B. dorei	Non	0	0	0	0	0	0
GCF_014131755.1_ASM1413175v1_protein	B. coprocola	Unknown	0	0	0	0	0	1

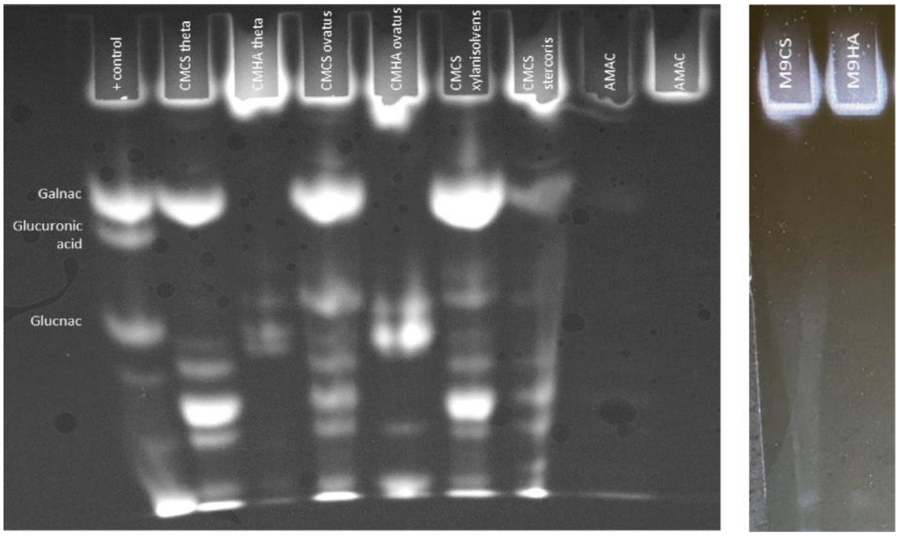

Supplementary figure 1: Representative FACE image for CS and HA degradation

Positive control contains GalNAc, GlcNAc and Glucuronic Acid. Samples are filtered media from degraders grown on either CS or HA. 2-Amino-9(10H)-acridinone (AMAC) was used as a negative control.

Supplementary table 7: Degradation of CS and/or HA by selected *Bacteroides* strains as deduced by FACE

		CS			HA		
	**Degrader**	**GalNAc**	**GlcA**	**Other bands**	**GlcNAc**	**GlcA**	**Other bands**
Cellulosilyticus	CS	Strong	faint	yes			
Intestinalis	CS	strong		yes			
Oleiciplenus	CS	strong	medium	yes			
Stercoris	CS	Strong	medium	yes			
Clarus	CSHA	Strong	faint	yes	medium	medium	yes, multiple
Ovatus	CSHA	Strong	medium	yes	strong		yes, 1
Theta	CSHA	Strong		yes	faint	faint	
Xylanisolvens	CSHA	Strong	faint	yes	n/a	n/a	n/a
Caccae	CS-HA	Strong		yes	strong		yes, 1

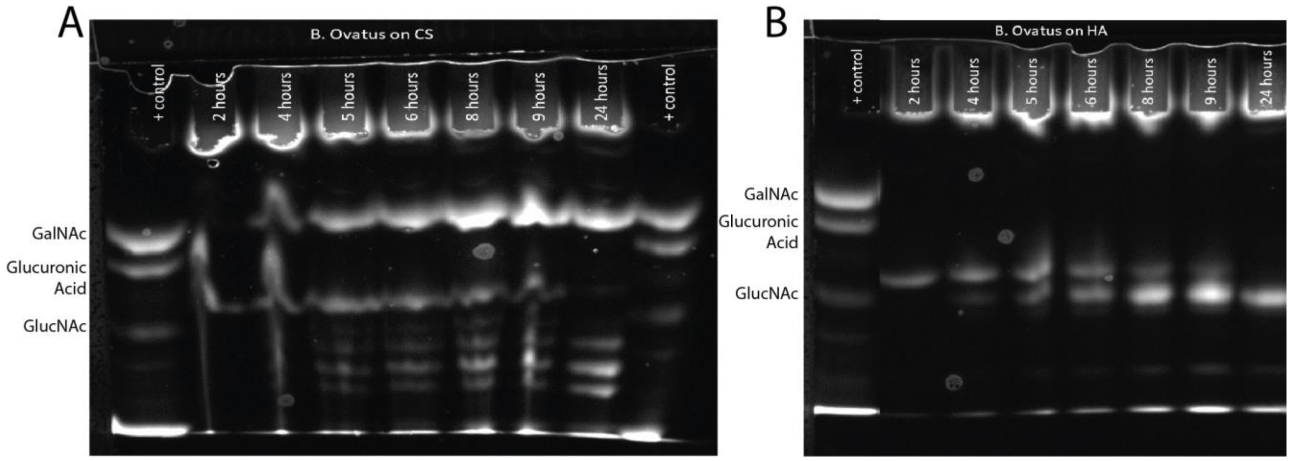

Supplementary figure 2: FACE image for *B. ovatus* grown on CS and HA sampled on multiple time-points.

A) Growth of *B. ovatus* on CS. After 4 hours, bands are already observed, with strongest GalNAc bands appearing between 8-9 hours. There are many other saccharides observed besides the two monosaccharides. B) Growth of *B. ovatus* on HA. First bands show after 2 hours, GlcNAc bands start showing between 4-5 hours. After 24 hours it seems only GlcNAc is left in the medium. Positive control contains GalNAc, GlcNAc and Glucuronic Acid. AMAC is a negative control.
